# Sequelæ of Typhoid Fever

**Published:** 1898-01-15

**Authors:** A. Maude

**Affiliations:** Medical Officer, Charing Cross Convalescent Home, Limpsfield, Surrey


### Medical Progress and Hospital Clinics.

[*The Editor will be glad to receive offers of co-operation and contributions from members of the profession. All letters should be addressed to* The Editor, at the Office, 28 & 29, Southampton Street, Strand, London, W.C.]

### SEQUELÆ OF TYPHOID FEVER.

By A. Maude, Medical Officer, Charing Cross Convalescent Home, Limpsfield, Surrey.

The sequelæ of no disease have attracted more attention than those of enteric. Chiefly from the fact that no medical ailment, no acute specific infectious fever except syphilis, presents so many surgical complications. In all writings and papers on convalescence the sequelæ of typhoid take up nearly half the space.

In Sir Dyce Duckworth's book,[1] he admits that during convalescence there may be recurrences of pyrexia, without any assignable causation, which simulate relapses of enteric. Such recrudescence of fever has been found, post-mortem, in several instances to be due to miliary abscesses in the kidneys. Such abscesses may be caused by streptococcus invasion; but in one instance of Osler's no other bacilli, except that of Eberth, was found. It is reasonable to assume that similar septic invasion may take place from late-healing intestinal ulcers, or from purulent foci in the spleen or mesenteric glands.

There is, however, no question that very late relapses of true enteric may occur.

W. F. began to ail at the beginning of September, 1896. He entered hospital on September 24th. By October 7th his temperature was normal, and he began solid food. On November 2nd he had a short relapse of three days' fever. On November 30th the temperature began to rise gradually, and fell gradually, with a chart exactly resembling a typical typhoid chart of three weeks' duration, normal being reached only on December 18th. This attack was accompanied from the third day of the rise by a copious outcrop of typical typhoid spots. There was no diarrhœa during either stage of his illness. The case has additional interest from the fact that there was, humanly speaking, no possibility of any reinfection. In the table given by Murchison[2] there are numerous instances of relapse occurring after 14 or 15 days' intermission of normal temperature, and I know cases where the intermission has been 18, 19, and 25 days. I have notes also of a second attack of typhoid nine weeks after the first, but this may have been due to reinfection, as in Goltdammer's case.

Maclagan maintains that late relapses are only met with when there has been constipation during convalescence, and according to Liebermeister, and others, relapses are more frequent after treatment by cold bathing. As in my case, when relapse takes place the eruption appears earlier than in the initial fever.

On Osler's authority we must note that in some instances there is a continued fever of low range (99°—101°) lasting two or three weeks, due apparently to the profound anæmia sequent to the acute illness; we have seen a similar rise of temperature occur in the anæmia produced by gastric ulcer with hæmorrhage.

We must distinguish carefully between true relapse and the occurrence of rigors and short pyrexia (sometimes of considerable intensity), which not unfrequently occur during lysis or in the post-febrile period. It is impossible to account fully for such rigors. They are sometimes accompanied by abdominal pains, and seem to be evoked by enemata or by straining at stool. The only reason for refusing to assign such rigors to septic infection of imperfectly healed patches is that there have in most cases been no clinical symptoms of ulceration at all. Possibly (as Dr. Herringham suggests) they are due to an irritability of thermic centres, produced by the original poison.

Delirium or mania during convalescence has been described in four cases by Andrewes.[3] The type of delirium is quite distinct from that of the acute form, and is more or less maniacal in character. Two forms may be recognised—(1) associated with collapse, and transient in duration, as described by Hermann Weber[4]; (2) a more permanent form, associated with inanition and anæmia. A large proportion of all the cases have been drunkards.

Any psychosis of ordinary type may supervene on enteric fever, mania, melancholia, hemiplegia with aphasia, and with optic atrophy (Leyden). Many patients, after severe attacks, remain speechless for long periods (35 days, Duckworth; 54 days, Church); they are not aphasic, or amnesic, or aphonic; they are intelligent, but quite silent. The writer has seen the same condition follow profound traumatic shock. Dr. Church's patient presented right hemiplegia, suggestive of localised cortical lesion. In other instances, this speechless stupor is probably a manifestation of general cortical exhaustion. This speechless condition, according to Handford,[5] is particularly common in children. I have myself seen it in a girl of seventeen, combined with paraplegia and paralysis of bladder, lasting many weeks. These cases, as far as is known, always recover.

A more or less chronic labyrinthine deafness may follow typhoid. The cases which recover are probably due to mere hyperæmia, while persistent deafness may be due to small cell infiltration of the labyrinth.[6]

Hartmann and Politzer ascribe as many cases of acquired deaf-mutism to typhoid as to scarlatina. In the chronic deafness of typhoid leeching over the mastoid should be employed (McBride); it can, at least, do no harm.

According to Marie,[7] the infectious fevers are the most important factor in the etiology of disseminated sclerosis, hence the undoubted fact that this nerve change never appears after forty years of age. Out of 25 cases of such origin the cause was typhoid. The influence of the fever is somewhat obscure, for if the micro-organism were the direct cause, we should expect to find disseminated sclerosis a much more common sequela than it is. Though it is impossible to come to any definite conclusion, Marie thinks that perhaps a mixed infection may be at work; this may perhaps account for the undoubtedly high percentage of cases due to typhoid and small-pox, in both of which a septic invasion may be added to the specific fever.[8]

The symptoms of insular sclerosis frequently do not develop till a year or more after the acute symptoms have all subsided.

Paralysis of cranial nerves is very uncommon. Optic neuritis is a very rare sequel. Paralysis of the third nerve was seen by Murchison[9] in a girl of 18, which cleared up, leaving only a slight ptosis; the nerve, or its centre, had, however, been similarly affected 14 years before in an attack of measles.

Only one case[10] of paralysis of accommodation (that of Gubler) is on record. This had been preceded by palatal paresis, and the inference is strong that diphtheritic infection had followed typhoid.

Paralysis of the vocal cords is rare, but has in two instances been so extreme as to necessitate tracheotomy. George Ross, however, published a case of multiple neuritis, in which he considered that diphtheria could be excluded, but palatal paresis and anæsthesia were marked.

It is worth noting that in only a small proportion of the large array of records given by Ross and Bury is alcohol excluded as a factor; but when we consider the enormous doses of spirits often ordered in typhoid this becomes a point of moment. A young subject, particularly a girl, who takes six or eight ounces of brandy a day for several weeks may easily present a peripheral neuritis which is not post-enteric but alcoholic.

Partial or even complete paraplegia is no uncommon effect of typhoid, both during the duration of fever and during convalescence.

Every degree of myelitis may exist; complete paraplegia is rare, but paresis of the legs, accompanied by spinal tenderness, and hyperæsthesia or other subjective symptoms in the legs, followed by diminution of sensation, is by no means uncommon. This may persist for many weeks, and may be accompanied by excessive knee jerk and ankle-clonus. Exaggeration of reflex and ankle clonus, persisting for many weeks, may even be found without paresis; but the rule (in my own experience) is that the knee jerks entirely disappear in later convalescence, though there is no paresis, and this is particularly common in children.

Gibney has described a condition to which American neurologists (who are fond of such names) have dubbed the "typhoid spine," characterised by pain radiating round the hips and abdomen from the spinal column, associated with general symptoms resembling neurasthenia; it occurs late in convalescence, and seems always to end in recovery.[11] Pepper considered it probably due to a periostitis of the front of the spinal column.

Poliomyelitis follows typhoid more frequently than any other specific fevers. To anterior poliomyelitis Gowers[12] ascribes the atrophic paralysis affecting groups of muscles which sometimes occur during convalescence. Paresis, rapid wasting, and loss of faradic irritability are present and may have permanent effects. An interesting case of this nature, in which wasting palsy of the arms was found (p.-m.) to be due to anterior poliomyelitis, is described by Shore.

Both Gowers and Duckworth discuss the difficulty of distinguishing such cerebral changes from a pure peripheral neuritis, but their remarks were made prior to our present knowledge of the relations of the grey matter cells to the periphery of the neuron.

A few instances of acute ascending paralysis of Landry's type have been known. (Gowers.)

With these cases the majority of paralyses following typhoid have been relegated by Ross and Bury to the class of peripheral neuritis.

A large proportion of patients complain of marked pain and tenderness in the distribution of the larger nerve trunks in the limbs. Tingling, numbness, and other disturbances of sensation, local asphyxia (lividity and coldness) of extremities often accompanying pain, and all these symptoms frequently herald a partial paralysis. This paresis is, as a rule, limited to certain muscles or groups of muscles, and often to the distribution of one particular nerve. There is no question, according to my observation, that certain nerve trunks are most often affected, viz., the ulnar, and the external and internal poplietal. This confirms the supposition advanced by Gower and Handford for the explanation of many cases that they are due to local injury, pressure, or stretching of the nerve trunks. Undoubtedly in any toxic state which may produce peripheral neuritis a slight pressure injury may produce a profound effect, but even when the brunt has fallen on one nerve there are usually signs of more widely-distributed disturbance. (Ross and Bury.)

Wasting of muscles invariably attends the paresis, and they show more or less electrical action of degeneration.

Sir D. Duckworth reports the occurrence of a specific (typhoidal) neuritis producing linear atrophy of the skin, accompanied by extreme hyperæsthesia of adjacent parts. The condition appeared three months after a severe enteric fever.

A highly interesting history was recorded by Shore,[13] in which muscular atrophy and paralysis of the upper limbs followed four months after an attack of typhoid, and fatal gangrene of the lungs supervened. The spinal cord was carefully examined, and found to present the appearance of a localised sub-acute anterior poliomyelitis of the cervical region, a condition corresponding entirely with the clinical symptoms. Criticising the condition by the light of Gaskell's researches on the incurvation of the viscera, Dr. Shore considers that the change in the spinal cord fully accounts for the gangrene of the lung, while there was no other cause present such as usually produces gangrene (embolism, &c.)

T. M. Simpson[14] records the history of a case in which typhoid proved the exciting cause of a hystero-catalepsy lasting off and on for many years, together with partial aphasia. Simpson refers to other cases in which cerebral weakness, imbecility, &c., have followed an attack of typhoid.

On the surgical sequelæ it is not necessary to dwell, as they are better known than those coming under the care of the physician, owing to the attention bestowed upon them by Paget, Keen,[15] and others.

Phlebitis is among the commonest and earliest sequences. The femoral vein, especially the left, is most usually affected. The pelvic veins are sometimes involved. Sudden pain in the pelvis, penis, bladder irritation, and smoky urine were the heralds of an evident thrombus in the internal pudic vein in a patient of Dr. Abercrombie's.[17]

Considering the frequency of thrombosis it is remarkable that the results from it are transitory and trifling compared with other thromboses; probably, however, the rest necessitated by the patient's general illness renders recovery more certain. For complete recovery is the rule, and detachment of clot is (as far as I am aware, says Duckworth) unreported. Moreover, suppuration never results in these thrombi, a fact which may be accepted in favour of their "mechanical" rather than septic origin.

Paget states that he had not seen after typhoid any corresponding number of cases of enlarged glands, diseased joints or ears, such as may follow other acute diseases, notably scarlet fever. Such post-enteric conditions of diseased bones and joints appear to be rare in England, but the literature of the subject shows that they are by no means rare in France and America.[18] Men suffer more than women from post-enteric bone lesions. Periostitis and perichonditis may manifest themselves late in convalescence. The tibia is the bone most frequently attacked. The rib next in frequency, then the femur, ulna, and parietal bones.

The extent of periostitis is usually small, and the area of necrosis, if this follows, smaller still. This fact, and that the accident frequently occurs in persons showing no scrofulous tendency, suggests that the causation is a thrombus.

Charcot pointed out that simple inflammation of the thyroid is a common sequel of enteric fever (as of rheumatism and small-pox). It cannot be very common, as I have made a routine practice of examining the gland in all typhoid convalescents, and have only once found it enlarged, in a boy of eight the subject of Graves' disease. The latter condition occasionally dates its manifestation from an attack of typhoid, but no one has established a connection between this and the acute specific thyroiditis.

Perichondritis of the larynx, with or without necrosis of the cartilages, is rather a late complication than a sequela.

We have as yet no certain evidence that many of the sequlæ we have noticed are due to the specific bacillus of Eberth. Many of the results are undoubtedly due (as in scarlet fever and diphtheria) to invasion by other organisms, the bacillus coli communis, streptococci, and staphylococci of the "septic" groups.

[1] Duckworth, Sequels of Disease (reprinted Lumleian Lectures). [2] Murchison, Continued Fevers, p. 553. [3] Andrewes, St. Barth. Hosp. Rep., XXV., p. 129. [4] Weber, Medico Chir. Soc., Trans. XL., viii. [5] Handford, Brit. Med. Journ., 1895, II., 705. [6] McBride, Diseases of Throat and Ear, p. 615. [7] Les Maladies de la Moelle, 1892. [8] Brain, 1896, p. 388. [9] Murchison, Op. cit., p. 563. [10] Ross and Burv, Peripheral Neuritis, p. 264. [11] Osler, Johns Hopkins Hosp. Rep., Vo. VII. [12] Gowers, Diseases of Nervous System, II., p. 834. [13] Shore, St. Bartholomew's Reps., XXIII., p. 114. [14] Edin. Med. Journ., Jan. 1897. [15] Paget, Clinical Lectures and Essays, 1897, p. 345. [16] Keen, Trans. Smithsonian Inst., 1877. [17] Chantmesse, Soc. Méd, des Hôp., July, 1890. [18] Abercrombie, Medico. Chirurg. Tran., LXXX., 1890. [19] Revue de Chirurgie., Sept., 1890.

